# Early *Salmonella* Typhimurium infection in pigs disrupts Microbiome composition and functionality principally at the ileum mucosa

**DOI:** 10.1038/s41598-018-26083-3

**Published:** 2018-05-17

**Authors:** Héctor Argüello, Jordi Estellé, Sara Zaldívar-López, Ángeles Jiménez-Marín, Ana Carvajal, Mª Asunción López-Bascón, Fiona Crispie, Orla O’Sullivan, Paul D. Cotter, Feliciano Priego-Capote, Luis Morera, Juan J. Garrido

**Affiliations:** 1 Grupo de Genómica y Mejora Animal, Departamento de Genética, Facultad de Veterinaria, Universidad de Córdoba, 14047 Córdoba, Spain; 2 GABI, INRA, AgroParisTech, Université Paris-Saclay, 78350 Jouy-en-Josas, France; 3 Departamento de Sanidad Animal, Facultad de Veterinaria, Universidad de León, 24071 León, Spain; 4 Departamento de Química Analítica Universidad de Córdoba, Córdoba, CeiA3 Campus de Excelencia Agroalimentaria, Universidad de Córdoba, 14047 Córdoba, Spain; 5 Teagasc, Food Research Centre, Moorepark, Fermoy, Co., Cork, Ireland; 6 APC Microbiome Institute, Cork, Ireland

## Abstract

*Salmonella* is a major foodborne pathogen which successfully infects animal species for human consumption such as swine. The pathogen has a battery of virulence factors which it uses to colonise and persist within the host. The host microbiota may play a role in resistance to, and may also be indirectly responsible from some of the consequences of, *Salmonella* infection. To investigate this, we used 16S rRNA metagenomic sequencing to determine the changes in the gut microbiota of pigs in response to infection by *Salmonella* Typhimurium at three locations: ileum mucosa, ileum content and faeces. Early infection (2 days post-infection) impacted on the microbiome diversity at the mucosa, reflected in a decrease in representatives of the generally regarded as desirable genera (i.e., *Bifidobacterium* and *Lactobacillu*s). Severe damage in the epithelium of the ileum mucosa correlated with an increase in synergistic (with respect to *Salmonella* infection; *Akkermansia*) or opportunistically pathogenic bacteria (*Citrobacter*) and a depletion in anaerobic bacteria (*Clostridium spp*., *Ruminococcus*, *or Dialliser*). Predictive functional analysis, together with metabolomic analysis revealed changes in glucose and lipid metabolism in infected pigs. The observed changes in commensal healthy microbiota, including the growth of synergistic or potentially pathogenic bacteria and depletion of beneficial or competing bacteria, could contribute to the pathogen’s ability to colonize the gut successfully. The findings from this study could be used to form the basis for further research aimed at creating intervention strategies to mitigate the effects of *Salmonella* infection.

## Introduction

*Salmonella* is a major foodborne pathogen with a significant number of cases and outbreaks every year^1^. Poultry meat, eggs and pork are the main sources of human salmonellosis. As a consequence of the successful establishment of control programmes in poultry, the relative proportion of human cases attributed to pork consumption has increased^2^.

Swine are a natural host for *Salmonella spp*. Apart from infections caused by *S*. Cholerasuis, the host adapted serovar to swine, *Salmonella* infections in pigs usually vary from subclinical to a self-limiting diarrhoea^3^. Among serovars affecting swine, *Salmonella* Typhimurium stands out due to its zoonotic importance in human salmonellosis^1^ and its widespread distribution in pork production^4,5^. The relevance of *S*. Typhimurium infections in swine is reflected by the vast literature published with respect to the pathogenesis^6^, the host immune response^7,8^, epidemiology of infections^3^ and control^9,10^ of this serotype in pigs. Notably, it has been reported that within a few hours of ingestion, the pathogen is detected in high concentrations in faeces^11^. Early after infection, epithelial damage and inflammation can be observed, principally at the ileum mucosa^12^, together with a migration of the pathogen to the gut associated lymphoid tissue via monocyte-derived cells^13^.

High throughput sequencing methods have provided an opportunity to study the porcine microbiota with unprecedented detail through sequencing of the 16S rRNA gene as a phylogenetic marker^14–16^. Indeed, these high throughput sequencing methods offer the chance to understand how infections such as swine salmonellosis impact the gut microbiota. For instance, this approach has been employed to identify differences in the microbiota among high and low *Salmonella* shedders^17^. In their study, Bearson and colleagues revealed a higher abundance of representatives of the family *Ruminococcaceae* in low-shedder pigs and also depletion of *Prevotella* in high shedder pigs early after infection. Another recent study, where pigs were challenged with *S*. Typhimurium, showed that strain virulence and the resulting intensity of the inflammatory response following infection by *Salmonella* caused a reduction in the abundance of short-chain fatty acids-producing bacteria^18^.

Understanding the changes occurring in the host microbiome due to *Salmonella* infection is of paramount importance with respect to developing a better understanding of *Salmonella* pathogenesis and disease. Here we characterise for the first time how the gut microbiome is modified after a *Salmonella* Typhimurium challenge in recently weaned pigs within three target locations of the gastrointestinal tract, i.e., faeces, ileum content and ileum mucosa. By studying changes in the diversity, taxonomy and abundance, and by combining this information with the predicted functionality of the microbiome and metabolite analysis, we aimed to expand on previous studies^17–19^, and provide a greater insight into the mechanisms underlying successful colonization of the gut by *Salmonella*^17–19^. Ultimately, it is hoped that identification of the groups of bacteria affected by disruption of gut homeostasis following *Salmonella* infection, could serve as a preliminary step to design strategies to limit the alteration of the microbiota by *Salmonella* infection and to increase resistance to gut colonization by this pathogen.

## Results

58014 operational taxonomic units (OTUs) were detected among the samples following matching of the 16S rRNA sequencing data to the Greengenes database. After filtering low abundance OTUs using a 0.005% threshold, a final number of 1192 taxa across seven taxonomic ranks were retained for further analysis.

### Factors affecting microbiome richness

**Figure 1:**
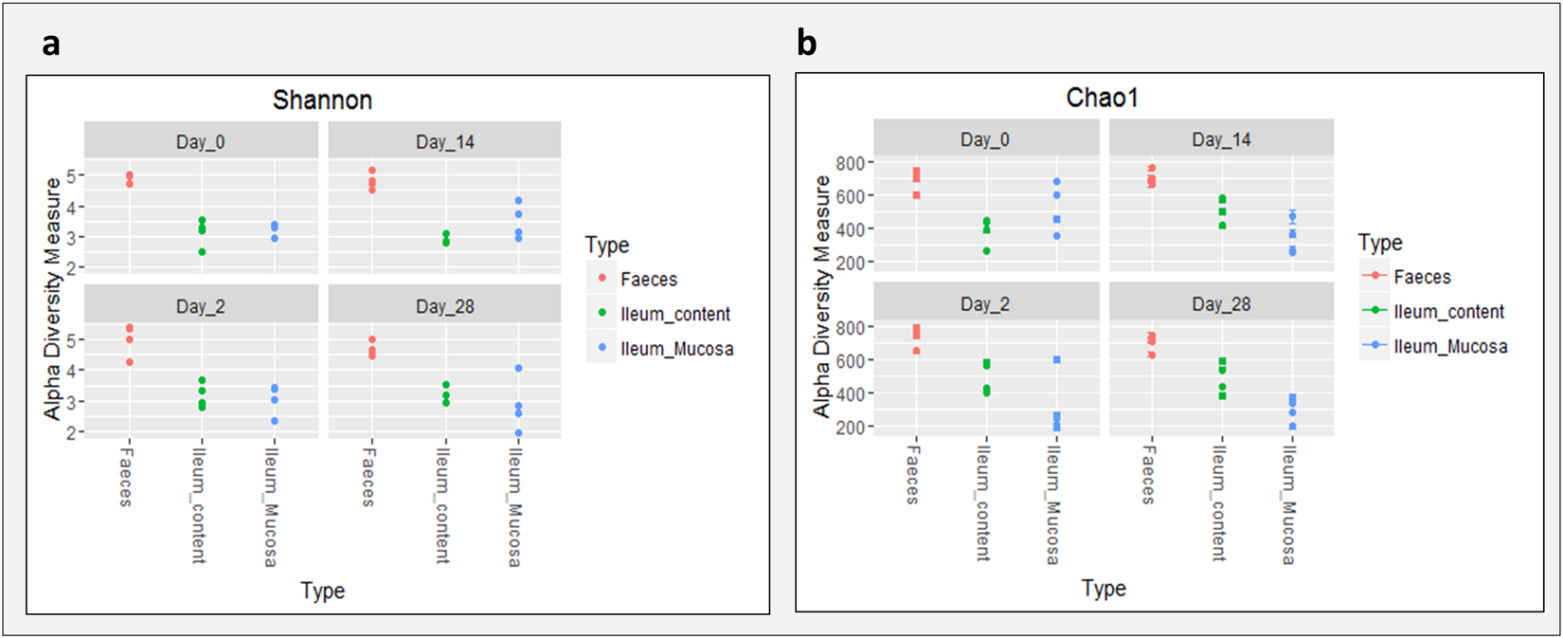
Estimation of eveness and richness (Shannon and Chao1) in faeces, ileum content and ileum mucosa samples during the time course of *S*. Typhimurium infection. Significant differences were observed regardless the estimator used when the type of sample factor was analysed (*p* < 0.001).

Sample richness was estimated by Chao1 and Shannon indices while Simpson index was used to estimate sample evenness (Fig. 1; Supplementary file 1). By repeated measure of analysis of variance (ANOVA), it was apparent that there were no significant differences in richness or evenness between infected and control pigs (*p* = 0.977; *p* = 0.75) or across sampling time points, (*p* = 0.96; *p* = 0.67). However, when ileum mucosa samples were specifically analysed, differences in richness among sampling time points were close to significance (Shannon index; *p* = 0.061). Across sample type, significant differences were observed in the richness estimators between faeces, ileum content and ileum mucosa (*p* < 0.001). Faecal samples showed higher richness than ileum content or ileum mucosa regardless of the estimator used (Fig. 1).

### Ordination of the pig gut microbiota varies according to the location in the gut from which samples are collected and between Day 0 and Day 2 samples

**Figure 2:**
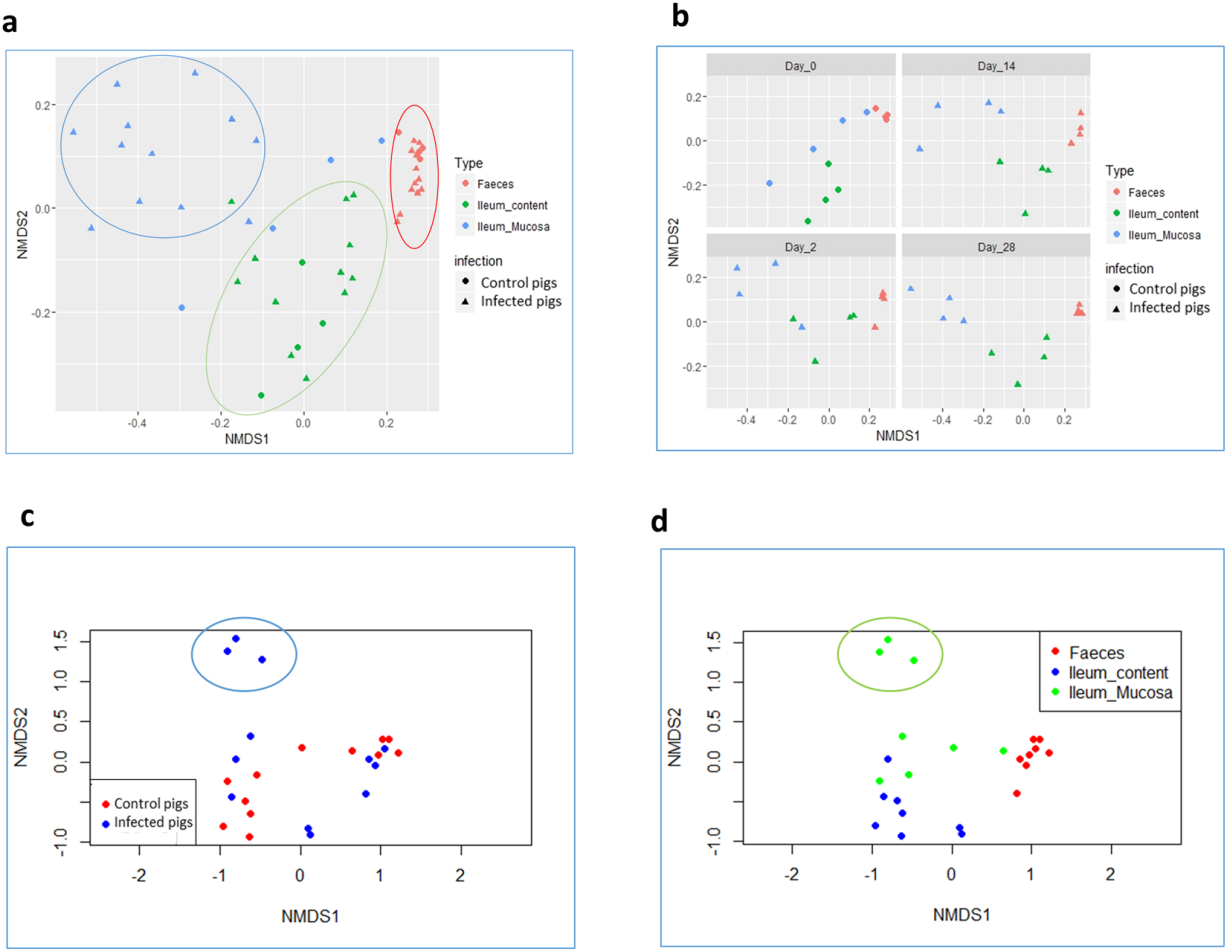
Ordination analysis of the samples obtained through the time-course of a *S*. Typhimurium infection in pigs, using Bray-Curtis dissimilarity index and plot by Non-metric Multidimensional Scaling (NMDS). (**a**) Ordination of samples by sample type factor (ileum content, ileum mucosa and faeces). (**b**) Splits the NMDS ordination by sampling day. (**c**,**d**) Include the ordination of samples from Day 0 (control pigs) and Day 2 (infected pigs). In (**c**) dots are coloured by infection factor, non-infected control pigs (red) and infected pigs at 2 days post-infection (dpi) (blue). (**d**) Represents the same ordination colouring the type of sample factor. See that ileum mucosa samples from 2 dpi pigs are positioned far from the rest of the samples green circle.

Two factors, i.e., type of sample (*p* < 0.001) and individual pig (*p* < 0.01), influenced the ordination of samples (Fig. 2a). The effect was maintained even after splitting samples by sampling day (Fig. 2b). Considering this output, the next ordination analyses were split by type of sample and the analysis of faeces, ileum mucosa and ileum content samples separately revealed that both infection (*p* < 0.01) and sampling day (*p* < 0.05) had a significant influence (Fig. 2). When samples from Day 0 (control group) and 2 days post-infection (d.p.i.) were specifically compared, significant differences in Bray-Curtis non-linear multi-dimensional scaling (NMDS) ordination were detected at within the ileum mucosa microbiota (*p* < 0.001) and close to significant differences (*p* = 0.053) were apparent in faecal samples (Fig. 2c and d).

### Taxonomic differences in faeces, ileum content and mucosa microbiomes at the time points evaluated

**Figure 3:**
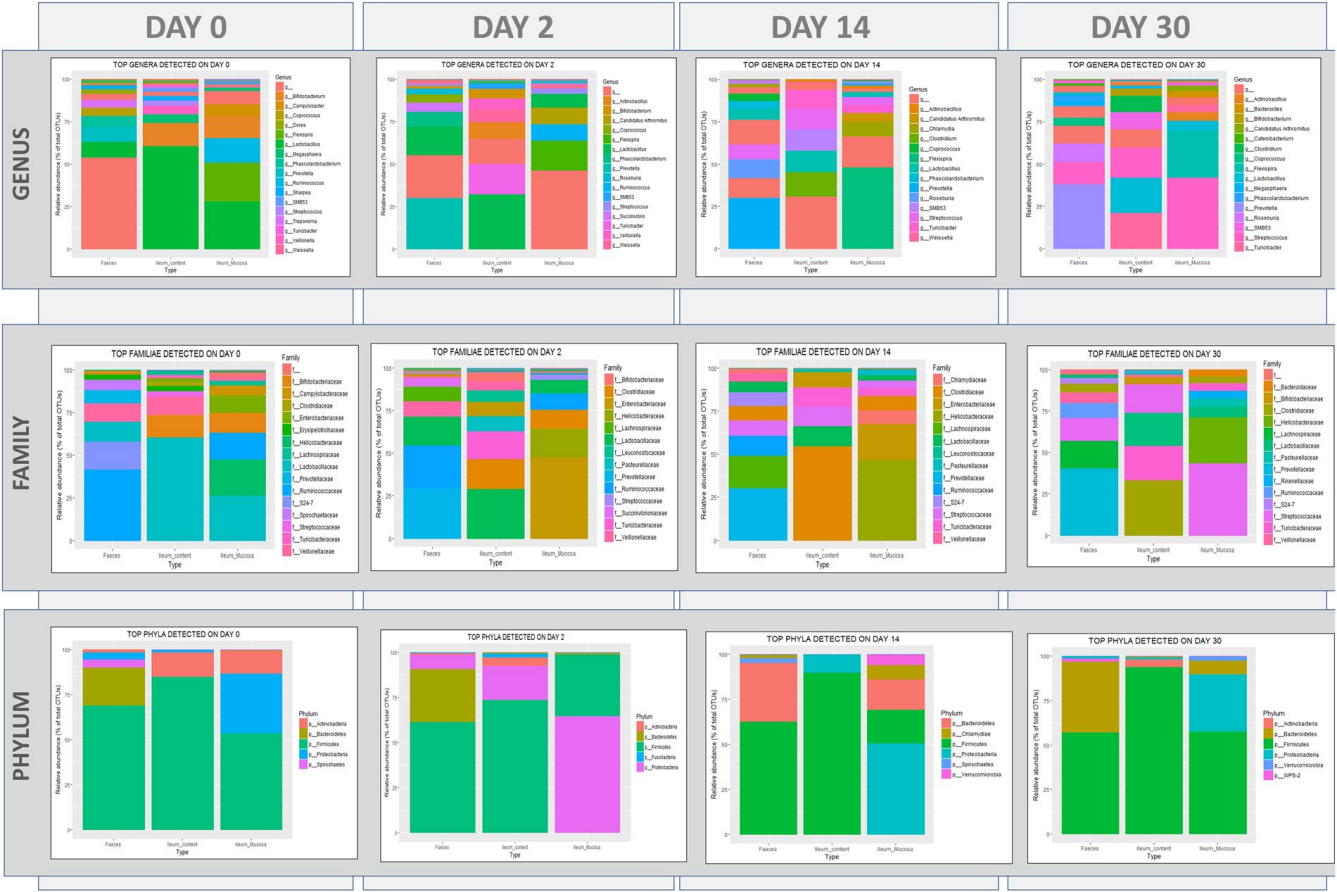
Relative percentage abundance of the main taxa (Phyla, Familiae and Genera) detected on each sampling day in each type of sample analysed (faeces, ileum content and ileum mucosa).

We compared the relative abundances of bacteria present in faeces, ileum content and ileum mucosa at different time points, including late infection sampling (14 d.p.i. and 30 d.p.i.; Fig. 3). OTUs were ranked by relative abundance, selecting up to the 10^th^ most abundant taxa (families and genera) in each sample type (faeces, ileum mucosa and ileum content) at each sampling point (Day 0, Day 2, Day 14 and Day 30). Overall, we observed large differences among samples across a variety of variables. On Day 0, *Lactobacillaceae* (*Lactobacillus*) dominated the bacteria present within the ileum contents. At the ileum mucosa *Lactobacillus*, *Helicobacteraceae* (*Flexispira*) and *Ruminococcaceae* (*Ruminococcus*) were the most abundant bacteria. In faeces, proportions of *Lactobacillaceae* were lower and the dominant family was *Ruminococcaceae*. Clear changes in dominant families and genera were observed in infected pigs at Days 2, 14 and 30 across intestinal locations. These differences were evident within ileum content and ileum mucosa samples, and are described in greater depth below. In contrast, the dominant families *Prevotellaceae* (*Prevotella*), *Lachnospirceae* (*Rosseburia* and *Coprococcus*) and *Ruminococcaceae* (*Ruminococcus*) remained relatively stable in faeces at different sampling points.

### Changes in the abundance of OTUs as a consequence of the *S*. Typhimurium infection

**Figure 4:**
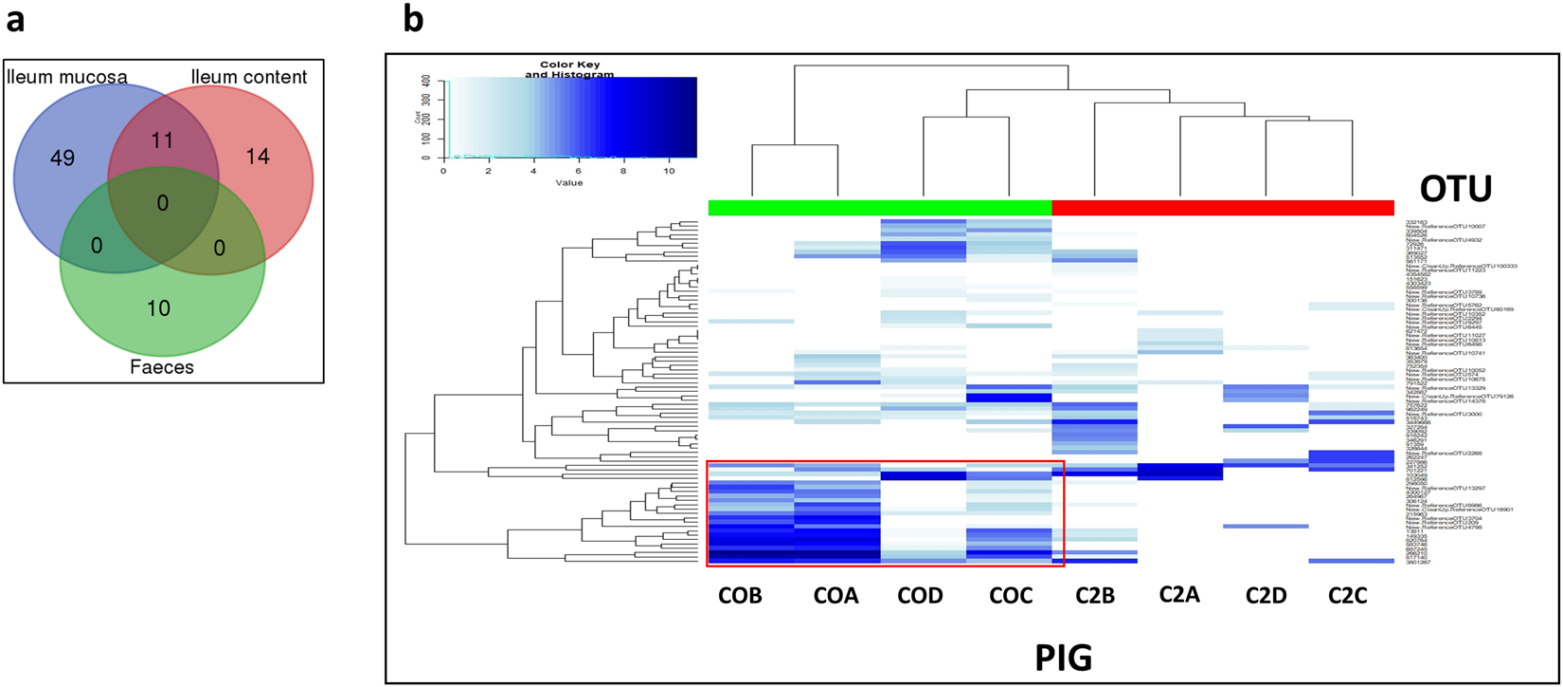
Differences in OTUs between control pigs (Day 0) and infected pigs (2 days post-infection) at the ileum mucosa, ileum content and faeces. (**a**) Venn diagram showing the overlapping of differentially expressed OTUs in ileum mucosa, ileum content and faeces between Day 0 and Day 2 p.i., pigs. (**b**) Heatmap illustrating the mean relative abundance of multiple OTUs at the ileum mucosa of non-infected control pigs (green) and Day 2 p.i. infected pigs (red). White colour indicates low abundance while dark blue high values of abundance. Dendrogram was built using hierarchical cluster analysis with Bray-Curtis dissimilarity indices. OTUs in the area included in the red frame belong to the family *Veillonellaceae* (*Mitsuokella*, *Megasphaera*, and *Dialister* genera).

The analysis of changes in microbiota abundance at 2 d.p.i. revealed 60 differentially abundant OTUs (FDR-value < 0.05) in the ileum mucosa (Supplementary file 2), when compared with the corresponding samples from the control group (0 d.p.i.). Similarly, 25 and 10 OTUs were found to be differentially abundant (FDR-value < 0.05) across the ileum content (Supplementary file 3) and faecal samples (Supplementary file 4), respectively. Particular OTUs differed significantly in abundance across the gut regions analysed, as depicted in Fig. 4a. Table 1 summarises the principal increased and decreased OTUs in the ileum mucosa, ileum content and in faeces after two days of infection. Notably, a decrease in abundance of OTUs belonging to the class *Clostridia* was apparent across all three locations analysed. Specifically, genera such as *Clostridium* (FDR = 0.037) and *Dialister* (FDR = 0.002) in the ileum mucosa, *Megasphaera* (FDR = 0.017) and *Mitsuokella* (FDR = 0.035) in the ileum content and two OTUs from families *Lachnospiraeae* (FDR = 0.033) and *Ruminococcaceae* (FDR = 0.027) in faeces, were all present in decreased proportions after two days of infection. In addition, OTUs from the genus *Lactobacillus* were also decreased in the ileum mucosa and content samples (FDR = 0.007, FDR = 0.042; respectively) at this time point.

**Table 1:** Principal OTUs changed (FDR < 0.05) in faeces, ileum content and ileum mucosa samples between control pigs (Day 0) and *Salmonella* infected pigs (Day 2 post-infection).

	Phylum	Class	Family	Genus	Counts Control group^a^	Counts Infected group^a^	Scaling factor	Adjusted p-value^b^
FAECES	*Firmicutes*	*Clostridiales*	*Lachnospiraceae*	Unclassified	1055	153	5.59	0.027
	*Firmicutes*	*Clostridiales*	*Ruminococcaceae*	Unclassified	8601	821	4.26	0.033
	*Firmicutes*	*Erysipelotrichales*	*Erysipelotrichaceae*	*p-75-a5*	3874	141	2.28	0.018
	*Chlamydiae*	*Chlamydiales*	*Chlamydiaceae*	*Chlamydia*	1	1580	2.23	0.008
	*Firmicutes*	*Clostridiales*	*Ruminococcaceae*	Unclassified	3273	22	1.69	0.008
	*Firmicutes*	*Clostridiales*	*Ruminococcaceae*	Unclassified	22	2529	0.39	0.008
	*Firmicutes*	*Clostridiales*	*Mogibacteriaceae*	Unclassified	0	344	0.25	0.025
	*Proteobacteria*	*Tremblayales*	*Unclassified*	Unclassified	9	570	−0.52	0.040
ILEUM CONTENT	*Firmicutes*	*Lactobacillales*	*Lactobacillaceae*	*Lactobacillus mucosae*	474	24	9.46	0.042
	*Proteobacteria*	*Campylobacterales*	*Campylobacteraceae*	*Campylobacter*	267	2	9.14	0.036
	*Firmicutes*	*Lactobacillales*	*Streptococcaceae*	*Streptococcus*	707	3	8.53	0.020
	*Firmicutes*	*Clostridiales*	*Veillonellaceae*	*Megasphaera*	312	0	7.35	0.017
	*Firmicutes*	*Clostridiales*	*Veillonellaceae*	*Mitsuokella*	1210	66	5.96	0.035
	*Proteobacteria*	*Enterobacteriales*	*Enterobacteriaceae*	Unclassified	0	690	−2.93	0.027
ILEUM MUCOSA	*Firmicutes*	*Clostridiales*	*Lachnospiraceae*	*Clostridium*	688	74	3.93	0.041
	*Firmicutes*	*Clostridiales*	*Lachnospiraceae*	*Clostridium*	802	83	3.71	0.037
	*Firmicutes*	*Clostridiales*	*Veillonellaceae*	*Dialister*	764	0	3.35	0.002
	*Actinobacteria*	*Bifidobacteriales*	*Bifidobacteriaceae*	*Bifidobacterium*	6456	4	3.05	1.081E-06
	*Actinobacteria*	*Bifidobacteriales*	*Bifidobacteriaceae*	*Bifidobacterium*	3132	1	3.04	1.022E-05
	*Actinobacteria*	*Bifidobacteriales*	*Bifidobacteriaceae*	*Bifidobacterium*	1767	3	3.02	1.565E-04
	*Actinobacteria*	*Bifidobacteriales*	*Bifidobacteriaceae*	*Bifidobacterium*	669	2	2.86	0.002
	*Actinobacteria*	*Bifidobacteriales*	*Bifidobacteriaceae*	*Bifidobacterium*	45161	38	2.67	1.695E-09
	*Firmicutes*	*Lactobacillales*	*Lactobacillaceae*	*Lactobacillus*	445	0	2.59	0.007
	*Verrucomicrobia*	*Verrucomicrobiales*	*Verrucomicrobiaceae*	*Akkermansia*	103	1691	−2.39	0.016
	*Firmicutes*	*Clostridiales*	*Ruminococcaceae*	*Oscillospira*	13	300	−2.84	0.032
	*Bacteroidetes*	*Bacteroidales*	*Odoribacteraceae*	*Odoribacter*	30	367	−4.51	0.035
	*Actinobacteria*	*Actinomycetales*	*Corynebacteriaceae*	*Corynebacterium*	4	537	−5.00	0.002
	*Firmicutes*	*Lactobacillales*	*Streptococcaceae*	*Streptococcus*	154	22415	−6.43	0.011
	*Firmicutes*	*Clostridiales*	*Clostridiaceae*	*Candidatus Arthromitus*	1	62633	−11.07	2.991E-08

^a^Normalised counts of OTUs.

^b^Adjusted p-value by false discovery rate.

The proportions of a number of taxa also increased at this time point (i.e., 2 dpi pigs). Genera such as *Candidatus arthromitus* (FDR < 0.001), *Akkermansia* (FDR = 0.016) and *Odoribacter* (FDR = 0.035) were increased within the mucosa of infected pigs, one OTU belonging to the *Enterobacteriaceae* family (genera unclassified) (FDR = 0.027) was significantly increased in ileum content samples and OTUs that were significantly increased in faeces were assigned to the families *Mogibacteriaceae* (FDR = 0.025) and *Ruminococcaceae* (FDR = 0.08).

Hierarchical clustering of pig samples according to the relative count of differentially abundant OTUs in the ileum mucosa, clustered pigs on the basis of infection (Fig. 4b). Samples collected from the four necropsied pigs at 2 d.p.i. (infected pigs) exhibited a similar pattern of OTUs abundance that diverged from the control pigs necropsied on Day 0. This clustering of pigs by the relative abundance of OTUs was not observed either in the ileum content or faeces for Day 0 and Day 2 pigs (Supplementary Figure S1a and b respectively).

### Correlation between histopathology scoring and mucosa-associated microbiota

**Figure 5:**
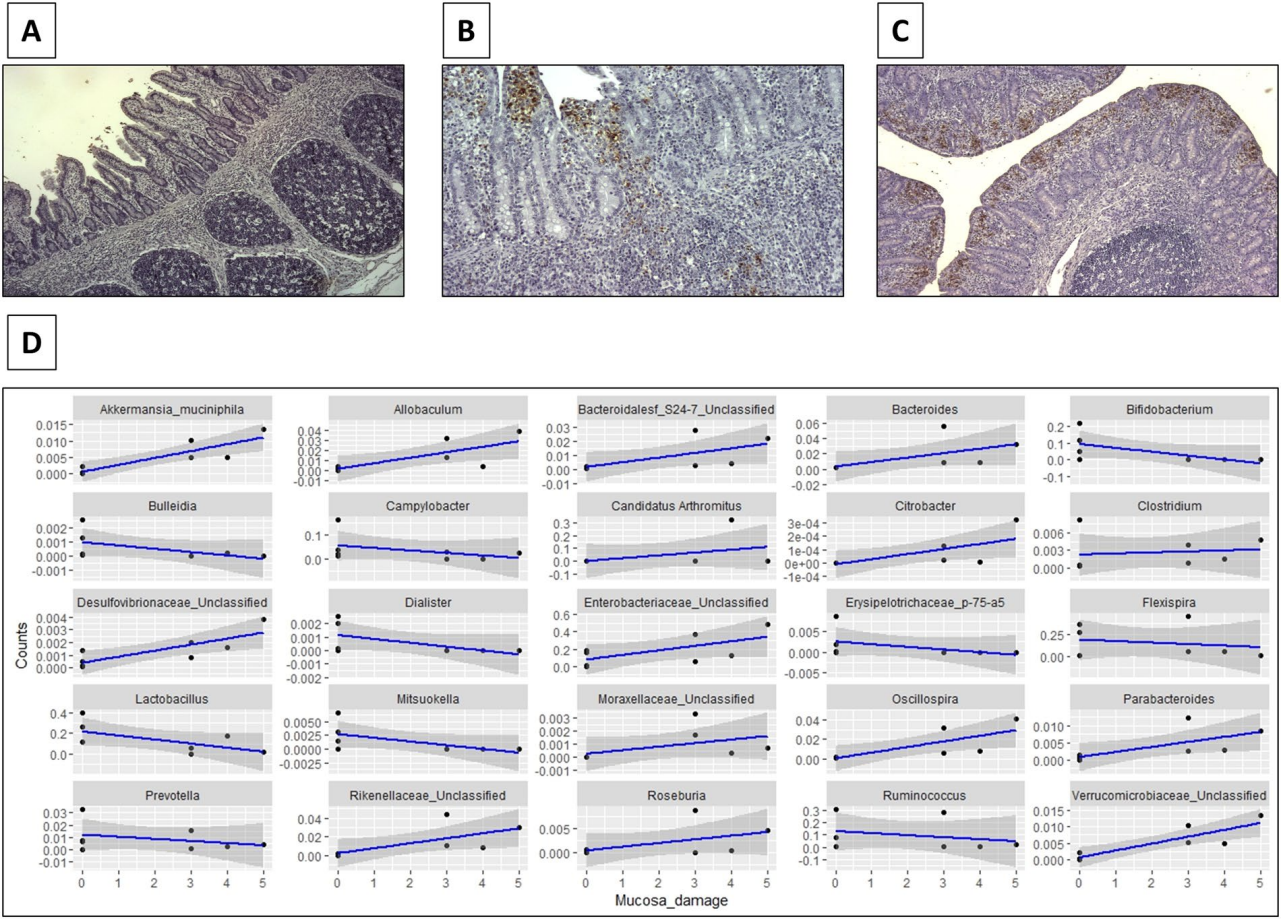
Genus correlated to integrity and damage of the epithelial of the mucosa. (**A**) Non-damaged mucosa from a control pig (score = 0), (**B**) damaged mucosa with villi shortened and *Salmonella* infiltration (score = 3) and (**C**) damaged mucosa with complete erosion of villi and high concentration of Salmonella (score = 5). (**D**) Relative counts of taxa correlated to epithelium damage at the ileum mucosa (*p* < 0.01).

Histological lesions in ileum mucosa samples from pigs necropsied on Day 0 and Day 2 were scored and correlated with OTU proportions at the mucosa (Fig. 5; Supplementary file 5). Severe epithelial damage was positively correlated to taxa from the phyla *Proteobacteria*, such as *Citrobacter* (Spearman’s *ρ* = 0.97 and *p* < 0.001), *Verrucomicrobia* such as *Akkermansia muciniphila* (Spearman’s *ρ* = 0.93 and *p* < 0.001), *Firmicutes*, such as *Allobaculum* (Spearman’s *ρ* = 0.92 and *p* < 0.001) or *Roseburia* (Spearman’s *ρ* = 0.88 and *p* < 0.01), and *Bacteroidetes*, such as *Bacteroides* (Spearman’s *ρ* = 0.87 and *p* < 0.01).

In contrast, the integrity of the intestinal epithelial cell wall was positively associated with different *Firmicutes*, such as *Lactobacillus* (Spearman’s *ρ* = −0.90 and *p* < 0.01), *Clostridium* (Spearman’s *ρ* = −0.88 and *p* < 0.01), *Bulleidia* (Spearman’s *ρ* = −0.87 and *p* < 0.01) and *Ruminococcus* (Spearman’s *ρ* = −0.87 and *p* < 0.01), *Proteobacteria*, such as *Campylobacter* (Spearman’s *ρ* = −0.88 and *p* < 0.01) and *Flexispira* (Spearman’s *ρ* = −0.87 and *p* < 0.01), and the genus *Bifidobacterium* (phylum *Actinobacteria*) (Spearman’s *ρ* = −0.88 and *p* < 0.01).

### Analysis of modulation of predicted microbiome functionality by *Salmonella* infection

The PICRUSt tool was used to predict Kegg Orthology group (KOs) abundances based on 16S rRNA sequences. The analysis yielded a prediction of 5441 KOs. As for OTU-based analysis, Bray-Curtis compositional dissimilarity highlighted significant differences in predicted functions (*p* < 0.001) by type of sample (faeces, ileum content or ileum mucosa (Supplementary Figure S2). Further analyses focused on differences between ileum mucosa samples from Day 0 and Day 2 pigs. No significant differences in metagenome functions were observed at KO level 2 functions although a tendency (*p* < 0.1) was observed in functions such as membrane transport or biosynthesis of secondary metabolites, all with lower abundance in infected pigs (Supplementary Figure S3). Analyses of functions at L3 in these samples identified 1138 differentially abundant KOs among control pigs (Day 0) and infected pigs (2 d.p.i) (Supplementary file 6). The inclusion of differentially expressed KOs in the software Interactive pathway explorer (Ipath2) showed an increased abundance of genes linked with the metabolism of lipids (biosynthesis of fatty acids) and nucleotide metabolism, and a decreased abundance of genes associated with bacterial chemotaxis, glycolysis and gluconeogenesis pathways in the ileum mucosa of Day 2 infected pigs.

Ileum content samples from control pigs (Day 0) and infected pigs (Day 2) were also analysed by liquid and gas chromatography. The analysis targeted fatty acids, fatty acyls and short-chain fatty acids compounds, carboxylic acid compounds and sugar derivatives. Table 2 summarises the 19 compounds that were significantly changed between control and infected pigs. Among fatty acids, there was a remarkable increase in concentration of dihydroxybutanoic (*p* = 0.026) acid, octanoic acid (*p* = 0.026) and acetoacetic acid (*p* = 0.002) in 2 dpi infected pigs. Among carboxylic acids, the levels of pyruvic (*p* = 0.002) and aconitic (*p* = 0.015) acids were significantly decreased in the ileum contents of infected pigs. Finally, glycerol, a sugar derivate, was also significantly decreased (*p* = 0.002) in the ileum content of Day 2 infected pigs.

**Table 2:** Chromatography table (mean normalised value) of differentiated metabolites between control pigs (Day 0) and *Salmonella* infected pigs (Day 2 post-infection) in ileum content samples.

Compound	Formula	Control group	Infected group (2 dpi)	*p*-value
**Fatty acids**, **fatty acils and short chain fatty acids derivates**				
Methylvaleric acid	C6H12O2	6765.50	10608.50	0.026
Oxovaleric acid	C5H8O3	1043565.50	6997420.00	0.0021
Acetoacetic acid	C4H6O3	56857.00	438702.50	0.0022
Dihydroxyisovaleric acid	C5HO4	412918.00	216793.50	0.0022
Dihydroxybutanoic acid	C4H8O2	182645.00	1595772.50	0.026
Octanoic acid	C8H16O2	196746.50	563696.00	0.026
Dihydroxy-docosanoic acid	C22H44O4	30227.16	5058.92	0.0021
Dihydroxystearic acid	C18H36O4	3918.66	314.47	0.0021
HODE	C18H32O3	19330.85	1830.28	0.0021
Hydroxyhexadecatetraenoic acid	C16H24O2	5224.16	5210.28	0.0086
Hydroxyoctadecenoic acid	C18H36O3	13607.09	17868.10	0.0259
Hydroxy tetracosanoic acid	C24H48O3	22198.33	4904.09	0.0259
Oxohexadecanoic acid	C16H30O3	280040.30	183366.84	0.0021
**Carboxilic acids**				
Aconitic acid	C6H6O6	54789.00	20940.50	0.0152
Pyruvic acid	C3H4O3	7913.00	2186.00	0.0022
Butenedioic acid	C4H4O4	95914.50	21361.00	0.0022
Pantothenic acid	C9H17NO5	1741.02	7536.10	0.0086
**Sugar derivates**				
Glycerol	**C3H8O3**	10788.00	3411.00	0.0022
Mannitol	C6H14O6	71854.00	505248.50	0.0022

## Discussion

This study involved an in-depth investigation of the microbial changes that occur in the gut environment at target locations, ileum content and mucosa as well as faeces, after a *S*. Typhimurium infection in swine. *S*. Typhimurium, either under controlled or field conditions, is able to succesfully infect pigs in a short time-frame, with relatively high concentrations of the pathogen being present in faeces within a few hours of exposure^11,20^. A number of studies agree that the local inflamation, resulting from the immune response elicited by *Salmonella*, triggers changes in the gut environment which favour the survival of *Salmonella* and prompt changes in the gut microbiota composition^18,21,22^. This inflamatory response is thought to enable *Salmonella* to compete with the resident microbiota through the use of tetrathionate produced by the oxidation of thiosulfate by reactive oxygen species (ROS)^23^ or due to its resistance to defensive molecules such as ROS or lipocalin-2, which is secreted by neutrophils and limits the acquisition of iron^24^. As a result, changes in the composition of the microbiota and gut functionality were anticipated. Our study included a limited number of pigs per sampling day. Although, ideally, a larger sample size would have been employed, our investigation with the limited number of pigs available, together with the appropiate statistical analysis, allow us to report, for the first time to our knowledge, that *Salmonella*’s biggest impact on microbiota composition takes places at the ileum mucosa as early as 2 d.p.i.

Methods of evaluating microbial diversity include α-diversity indexes such as richness (the total number of unique species within a community) and the relative abundance of different species that make up the richness in that community, and as well as β-diversity, dissimilarity-based ordinations. These metriceswere clearly influenced by the origin of the sample (ileum content, ileum mucosa or faeces) in accordance with previous results^25,26^. When samples were split by sample type, the influence of the infection on the ordination of the microbiota at the ileum mucosa was particulalrly apparent. In contrast, it was surprising to note that although challenged pigs exhibited clinical signs, such as mild to watery diarrhoea and low-grade fever, as well as the steady shedding of *Salmonella* during the first week (data not shown), no clear significant differences in diversity were observed in faeces (only a trend), or ileum content samples. Although the sample size limits the power to detect differences in diversity in faeces, our observation is consistent with the results obtained by Bearson and colleagues^17^, who observed that neither alpha nor beta diversity were affected when challenged pigs shed *Salmonella* at low concentrations in faeces. Furthemore, Drumo *et al*.^18^ showed that *Salmonella* strain virulence influences the observed changes of diversity results in faeces. In our results, we establish for the first time that the major diversity differences occur at the ileum mucosa and that this effect is less apparent within the ileum content or the downstream segments of the intestine (i.e., faeces).

In agreement with previous studies in pigs^17–19^, we found that *Salmonella* infection triggered changes in the relative abundance of certain bacteria at 2 d.p.i. The shift in the microbiota was apparent at the ileum mucosa (see dendogram in Fig. 4b), where samples from 2 d.p.i. infected pigs clustered together in the same clade, influenced by the drop in abundance of genera from the family *Veillonellaceae* (*Mitsuokella*, *Dialister* and *Megasphaera*). Since the ileum constitutes the principal target for *Salmonella* invasion in the gut^27^, it can be inferred from our results that the aforementioned impacts on epithelial cell damage, inflammation, defensive molecules and metabolites principally affect the microbiota associated to the ileum mucosa, while the effect on ileum content or faeces is more limited.

To evaluate the effect of the infection on the relative abundance of the microbiota, relative changes in taxa abundance between Day 0 and Day 2 were evaluated. In agreement with previous studies, we observed dissimilarities in OTU changes at different locations after *Salmonella* infection^19^. Although the microbiota is not stable and evolves through the pigs’ life^26^, the short time-frame between challenge and infection (2 days) ensures that the changes observed cannot be attributed to age-related influences. Notably, our results demonstrated a decrease in abundance of beneficial gut bacteria such as *Clostridium spp*., *Lactobacillus spp*., *Prevotella*, *Bifidobacterium* or *Megaesphera* in the ileum (mucosa and content) after infection. The decrease in *Lactobacillus* was one of the most robust results with respect to the microbiota of the ileum content and ileum mucosa, with a number of different OTUs of these species ranking among the most decreased. *S*. Typhimurium infection in pigs prompts an early inflamatory response which is associated with a decreased expressionof genes coding for apical sodium-dependent bile acid transporter (ASBT) and the fatty and bile acid transporters FABP2 and FABP6, impairing normal bile absorption in the ileum^8^. As a result, higher concentrations of bile salts are present in the lumen of the small intestine. As some beneficial species such as *Lactobacillus* and *Bifidobacterium*, are susceptible to bile salts^28,29^, the marked drop of abundance of these bacteria in the ileum could be the consequence of the disruption of the entero-hepatic circulation of the biliary acids. However, it remains to be seen if these changes are transitory, i.e., as a consequence of the challenge with a high dose of *Salmonella*, or if they persist for longer periods. It is notable that previous work does show that the changes in microbiome community structure persist over the first week post-challenge^17,19^.

We also observed taxa changes specific to each location, e.g., *Dialister* only changed at the mucosa, *Veillonella* at the ileum content and *Treponema* in faeces. Even if divergences in changes in the microbiota at different locations could be explained by the differences of the background microbiota^25^, we propose that a diluted effect in faeces compared to the ileum may also be a contributory factor. Through the analysis of the most abundant taxa in samples Day 14 and Day 30, it was apparent that the faecal microbiome was more stable throughout the course of the study than was the case for the ileal microbiomes^14^. This result highlights that the microbiome is more susceptible to changes in targeted locations (e.g. ileum mucosa) than in faeces (which can be considered a global picture of the gut microbiome), a fact that is particularly notable in light of the fact that faeces is regularly used in microbiome studies due to the fact that sample procurement is non-invasive^30^. Although our results may be limited by the study sample size, we illustrate that the interpretation of the results should be validated in the context of the anatomical location of the studied effect when faeces are used as proxy samples.

Damage of ileal mucosa was correlated to an increase of bacteria that are synergistic to the infectious process, i.e., bacteria that boost the damage caused by the pathogen, and/or opportunistic pathogens, such as *Akkermansia muciniphila* and *Citrobacter*, respectively. Previous studies have reported an increase in levels of other *Enterobacteriaceae*, such as *Citrobacter*, arising from *Salmonella* infection^22,31^. Our study confirms that the modification of the intestinal environment at the ileum favours the expansion of closely related microorganisms^22,32,33^. *Akkermansia* is an opportunistic bacterium which develops synergistic relationships with *Salmonella* by disturbing mucus secretion and silfation^19^. Similarly to our study, Borton *et al*.^22^ detected an increase in the proportions of the genus *Akkermansia* genera in *Salmonella* infection with inflamed murine gut samples. In contast, typically regarded as beneficial, anaerobic, Gram-positive gut bacteria such as *Lactobacillus*, *Ruminococcus*, *Clostridium* or *Allobaculum* abundance decreased in severe damaged ileum mucosa samples. The release of RNS and ROS during intestinal non-typhoidal *Salmonella* infection creates a highly oxidative environment which is not permissive for the growth of anaerobic bacteria^34^, while transitient aerotolerant bacteria such as *Bacteroides*^35^ might take an advantage of the disruption in the gut enviromental conditions.

Finally, based on PICRUSt predictions and metabolomic analysis, we inferred changes in functional pathways encoded within the ileum mucosa microbiome associated with infection. Ordination of predicted functions by Bray-Curtis dissimilarity clustered samples by tissue, similarly to 16S rRNA OTU results. At KEGG level 2, there were no clear differences in microbiome functions among control and early infected pigs at the ileum mucosa. The sample size together with the overlapping of functions among different bacteria are two possibly limiting factors in finding potential differences^30^. Nevertheless, some functions related to basal metabolism (secondary metabolism, metabolism of enzymes, amino acids and biosynthesis of lipids) demonstrated a tendency to be reduced at 2 d.p.i., an observation that suggests a general downshift in microbial intestinal activity. These results are in accordance with a study of induced colitis in pigs^36^, which described a reduced capacity for energy acquisition and dysregulated microbial signaling and repair pathways after inflammation. Further analysis of functions at KEGG level 3 interestingly revealed that chemotaxis and glucose metabolism (glycolysis and gluconeogenesis) functions were more abundant at the ileum mucosa prior to *Salmonella* infection (control pigs). The activation of the glucose metabolism could be associated with the degradation of oligo- and polysacharides by lactic acid bacteria. Levels of glycerol, a sugar derivative, and pyruvate, a carboxylic acid, were decreased in infected pigs, acording to the predictions in the metabolism of glucose and its derivatives. In contrast, levels of pantothenic acid were increased in the ileum contents of infected pigs at 2 d.p.i. Analysis of predicted metabolic routes in Interactive Pathways Explorer (Ipath2) showed an activation of valine, leucine and isoleucine metabolism, all of which result in the production of pantothenic acid. At 2 d.p.i., an increase of fatty acid metabolism KOs was observed, with particular activation of fatty acid biosynthesis routes. Levels of methylvaleric, oxovaleric, acetoacetic, butenedioic and octanoic acids were increased in ileum contents from 2 d.p.i. pigs, confirming the activation of biosynthesis of fatty acids by the gut microbiota early after infection. The link between the microbiota and fatty acid metabolism could be attributed to changes in the nutrients available in the gut environment. *Salmonella* infection disrupts the entero-hepatic circulation of the billiary acids as mentioned above^8,30^, which in turn alters the lipid metabolism. As a result, we can expect higher concentrations of available fatty acids in the gut lumen, favouring the growth of bacteria that use them in their metabolism. Overall, these results illustrate that *S*. Typhimurium infection triggers impairment of metabolic funtions, which in turn can affect the physiology of digestive processes in the gut^30^.

## Conclusions

The present study demonstrates that *Salmonella* infection modifies the microbiota in the gut early after infection, and shows for the first time that the major changes occur in the ileum mucosa and are less evident in the faeces and even the ileum lumen, highlighting the merits of study host mucosal samples, where practical. Ironically, host response to infection (immune response and metabolic changes) could be a major contributor to the depletion of commensal/beneficial inhabitants of the intestinal tract (*Lactobacillus*, *Bifidobacterium*, *Prevotella* or *Megasphaera*), and the increase of synergists of infection such as *Akkermansia* or *Citrobacter*. Indeed, the relative abundance of these synergists in the ileal microbial ecosystem is positively correlated to the degree of epitelium damage in the ileum. Finally, we illustrate that the disruption of microbiota composition triggered by infection also has apparent consequences for the microbiome function, with a general downshift of activities early after infection and additional changes in relevant metabolic routes. Future research should focus on identifying the key host response-related factors that prompt changes in the microbiome structure, which might help to establish novel microbiome restoration strategies to limit the impact of *Salmonella* infection in pigs and, ultimately, zoonotic infections in humans.

## Methods

### *Salmonella* challenge outline

Sixteen crossbred commercial weaned female pigs of approximately four weeks of age from a *Salmonella*-free herd were moved into the isolation facilities at the University of León (Spain). After an adaptation period of seven days, four pigs were euthanized and necropsied (control group). The remaining twelve pigs were orally challenged with 5 ml of a broth containing 10^8^ cfu/ml of *S*. Typhimurium phage type DT104 and subsequently necropsied in groups of four at 2 days post-infection (d.p.i.), 14 d.p.i., and 30 d.p.i.

Faeces and ileum (approx., 20 cm backwards from ileocaecal junction) were collected from each animal. Ileum luminal content (approx. 5 g) was poured in a plastic tube and the ileum wall was preserved in another tube. All samples were collected aseptically and frozen in liquid nitrogen immediately. Frozen samples were kept at −80 °C until further processing. A subsample of faeces was cultured by the current ISO:6579/2007 to detect the presence/absence of *Salmonella* in all pigs included in the study. Another sub-sample of ileum wall was fixed in formalin and embedded in paraffin wax for further processing.

All procedures involving animals were performed in accordance with the European regulations regarding the protection of animals used for experimental and other scientific purposes, under the supervision of the Ethical and Animal Welfare Committee of the University of León (Spain).

### Histopathology and immunohistochemistry

Two paraffin sections (5 μm) from each formalin fixed samples were routinely processed and stained with hematoxylin and eosin (H&E) to evaluate tissue morphology. For immunohistochemistry assays, a standard avidin-biotin peroxidase method was performed as described elsewhere^37^ employing 1F12 monoclonal antibody and a biotinylated anti-mouse IgG (Dako, Barcelona, Spain) as a secondary antibody. Sections were evaluated by microscopy and scored (from 0 to 5) by adding up the following parameters: no lesions = 0; villi shorten = 1; complete villi erosion = 1; presence of *Salmonella* = 1; high concentration of *Salmonella* = 1; inflammatory cells infiltration = 1.

### DNA extraction

Frozen ileum wall (5–10 cm of ileum) was opened using a scalpel, mucosa wall was rinsed carefully with sterile Phosphate Saline Buffer to remove any faecal matter and its surface was scraped without excessive pressure using a sterile slide.

Purelink Genomic DNA mini-kit (Invitrogen) was used to extract DNA from 200 mg ileum mucosa (IM) while Purelink Microbiome DNA Extraction kit (Invitrogen) was used for Ileum content (200 mg) and faeces (200 mg) samples. Both kits were used in accordance with manufacturer’s instructions. After extraction, DNA was quantified (Nanodrop®, ThermoFisher) and the presence of microbial DNA was confirmed by 16S rRNA amplification by conventional PCR^38^.

### 16S rRNA amplicon sequencing

All samples were prepared for MiSeq compositional sequencing using the specifications outlined by Illumina Inc. (Illumina Inc., Cambridge, UK). The V3–V4 region of the 16S rRNA gene was amplified and Illumina index primers attached in two separate PCR reactions^39^. All PCR reaction conditions and clean up procedures using AMPure XP (Labplan, Kildare, Ireland) were followed as outlined by Illumina Inc. Quantified samples were then sequenced using an Illumina MiSeq system at the Teagasc Sequencing Centre (Moorepark, Fermoy, Ireland).

### Bioinformatic processing and analysis

Raw sequence reads generated by MiSeq sequencing were processed using the version 1.9.1 of the Quantitative Insights Into Microbial Ecology (QIIME) pipeline^40^ by using the subsampled open-reference OTU calling approach^41^. Demultiplexing and trimming of Miseq reads was done with the default QIIME parameters^42^. After trimming, the reads were merged into a single fasta file and clustered into OTU against the GreenGenes database^43^ (release 2013-08: gg_13_8_otus) by using the parallel *uclust_ref* method. Reads that failed this step were clustered to *de novo* OTU with the *uclust*^44^ method. The filtering of chimeric OTU was performed by using ChimeraSlayer^45^ against the GreenGenes reference alignment. After removing singletons and doubletons OTUs, only those OTUs representing more than 0.005% of the total filtered were retained as suggested by Bokulich *et al*.^42^. For the genera analyses, OTU were collapsed into genera taxonomic level by using the tax_glom funciont in PhyloSeq^46^. The full data sets have been submitted to NCBI Sequence Read Archive (SRA) under accession: SRP111505, (BioProject:PRJNA393762).

### Functional metagenomic predictions

A functional prediction based on 16S rRNA marker gene sequences was performed using PICRUSt^47^. After excluding the unknown OTUs from the GreenGenes 13.5 reference database and normalizing by 16S rRNA gene copy number, functional metagenomes for each sample were predicted from the Kyoto Encyclopedia of Genes and Genomes (KEGG) catalogue and categorised to a specified KEGG level. In addition, Kegg orthology groups (KOs) were mapped to KEGG and visualized using the Interactive Pathway Explorer (iPath2.0) web-based tool^48^.

### Detection of metabolites by metabolomics

Detection of metabolites was performed from 160 mg of ileum content samples from Day 0 and Day 2 pigs by liquid chromatography time-of-flight mass spectrometry (LC–QTOF MS/MS) and Gas chromatography time-of-flight mass spectrometry (GC–QTOF MS). Prior to the analysis, an extraction of the metabolites was carried out with methanol and dichloromethane.

LC–QTOF MS/MS was run in Agilent 1200 Series LC system coupled to an Agilent 6540 UHD Accurate-Mass QTOF hybrid mass spectrometer (Santa Clara, CA, USA). Chromatographic separation was performed by using a Poroshell 120 EC-C18 column (50 mm × 2.1 mm i.d., 2.7 μm particle size) from Agilent Technologies. The mobile phases were 95:5 water:ACN (phase A) and 95:5 ACN:water (phase B), both containing 0.1% *(v/v*) formic acid and 5 mM ammonium acetate as ionization agents. The LC pump was programmed at a flow rate of 0.25 mL min^−1^ and the elution gradient was as follows: from min 0 to 30, the percentage of phase B was modified from 50% to 100%, and then the final percentage was held for 20 min. Thus, the total analysis time per sample was 61 min (including postprocessing). Accurate mass spectra in MS scan were acquired in the *m/z* range 40–1200. MassHunter Workstation software (version B7.00 Qualitative Analysis, Agilent Technologies, Santa Clara, CA, USA) was used to process all data obtained by LC–QTOF in auto MSMS mode. Identification of the metabolites was supported on MS and MS/MS information and search in the METLIN MS and MS/MS databases (http://metlin.scripps.edu), the Human Metabolome Database (HMDB, 3.6 version) and the LIPID MAPS website ((http://www.lipidmaps.org), using in all cases the MFs obtained in the previous step. A table with the peak area of all identified compounds in the different samples injected was obtained as a result.

GC–QTOF/MS analysis was performed by Agilent 7890 A Series GC system coupled to an Agilent 7200 UHD Accurate-Mass QTOF hybrid mass spectrometer equipped with an electron impact (EI) source (Santa Clara, CA, USA). The samples were analyzed by EI ionization mode at 70 eV. Chromatographic separation was carried out with a fused silica DB-5MS-UI 30 m × 0.25 mm i.d, × 0.25 μm film thickness capillary column. The GC oven temperature program started at 60 °C (1 min held), followed by a temperature ramp of 10 °C min^−1^ to final 300 °C (2 min held). Post-run time was programmed for 4 min up to 310 °C to assure complete elution of the injected sample. MassHunter Workstation software (version B7.00 Qualitative Analysis, Agilent Technologies, Santa Clara, CA, USA) was used to process all data obtained by GC–TOF/MS in full scan mode. The list of MFs obtained for each analysis was exported as data files in compound exchange format (.cef files). Tentative identification of compounds was performed by searching each mass spectrum in the NIST 11 and Fiehn databases using the retention index or retention time value, respectively.

### Statistical analysis

Further analyses were performed in R v3.1.1. Microbiota and study variables (type of sample, sampling, infection) were included in the estimation of alpha diversity (Shannon and Chao1 indexes) and beta diversity (Simpson index) analysis by Vegan R package^49^. Potential differences in richness of factors included in the study were estimated by analysis of variance (ANOVA). Dissimilarities between pairs of samples were estimated with the Bray-Curtis dissimilarity index^50^ and weighted unifrac index^51^ and analysed with non-linear multi-dimensional scaling (NMDS) in Vegan. The Vegan envfit function was used to evaluate if the factors of study (*i*.*e*. sampling day and infection status) where associated to the NMDS ordinations; the significance of the fitted factors was estimated by using 999 permutations.

Normalisation of OTUs counts is recommended for further statistical processing^52^. The metagenomeSeq R package^53^ was used for data normalisation and further statistical analysis of differences in OTUs abundance. By using the *fitZig* function, a zero-inflated Gaussian mixture model, OTUs counts were normalised by calculating the scaling factors equal to the sum of counts. The parameters for the mixture model were estimated with an expectation-maximization algorithm, coupled with a moderated *t* statistic. Individual pig was used as a covariate in the analyses. Differences in OTUs threshold was fixed at a false discovery rate (FDR) value of 0.05. Finally, KO abundance obtained from PICRUSt was introduced into R using PhyloSeq and statistical differences were estimated using Deseq 2^54^ using the Wald-test and a FDR threshold of 0.05. The relationship between histopathological lesions and abundance of bacteria in the ileum mucosa was explored by non-parametric Spearman’s rank correlation coefficient test implemented in Qiime.

Metabolites abundance were measured by the normalised area under the curve. For each metabolite, comparisons were made by the non-parametric Wilcoxon-test in R with a significant threshold of 0.05.

### Ethical approval

All procedures involving animals were approved by the institutional bioethical committee fo the University of Leon (license number ULE_003_2015) and performed according to European regulations regarding animal welfare and protection of animals used for experimental and other scientific purposes.

## Electronic supplementary material

Supplementary figures

Supplementary Dataset 1

Supplementary Dataset 2

Supplementary Dataset 3

Supplementary Dataset 4

Supplementary Dataset 5

Supplementary Dataset 6
