# Supplementary material for: Early Salmonella Typhimurium infection in pigs disrupts Microbiome composition and functionality principally at the ileum mucosa
Source: Sci Rep. 2018 May 17;8:7788. doi: 10.1038/s41598-018-26083-3 (PMC5958136; doi:10.1038/s41598-018-26083-3)
Supplement: Supplementary file 1 — Supplementary figures [file 41598_2018_26083_MOESM1_ESM.pdf]

Early Salmonella Typhimurium infection in pigs disrupts Microbiome composition and functionality principally at the ileum mucosa.

AUTHORS

Héctor Argüello[1*], Jordi Estellé[2], Sara Zaldívar-López[1], Ángeles Jiménez-Marín[1], Ana Carvajal[3], Mª Asunción López-Bascón[4], Fiona Crispie[5,6], Orla O'Sullivan[5,6], Paul D. Cotter[5,6], Feliciano Priego-Capote[4], Luis Morera[1], Juan J. Garrido[1].

INSTITUTIONS

[1]Grupo de Genómica y Mejora Animal, Departamento de Genética, Facultad de Veterinaria. Universidad de Córdoba, 14047, Córdoba, Spain.

[2]GABI, INRA, AgroParisTech, Université Paris-Saclay, 78350 Jouy-en-Josas, France.

[3]Departamento de Sanidad Animal, Facultad de Veterinaria, Universidad de León, 24071, León, Spain.

[4]Departamento de Química Analítica Universidad de Córdoba, Córdoba; CeiA3 Campus de Excelencia Agroalimentaria, Universidad de Córdoba, 14047, Córdoba, Spain.

[5]Teagasc, Food Research Centre, Moorepark, Fermoy, Co. Cork, Ireland.

[6]APC Microbiome Institute, Cork, Ireland.

EMAIL ADDRESSES:

* Corresponding author: Héctor Argüello: ge2arroh@uco.es

Figure S1a

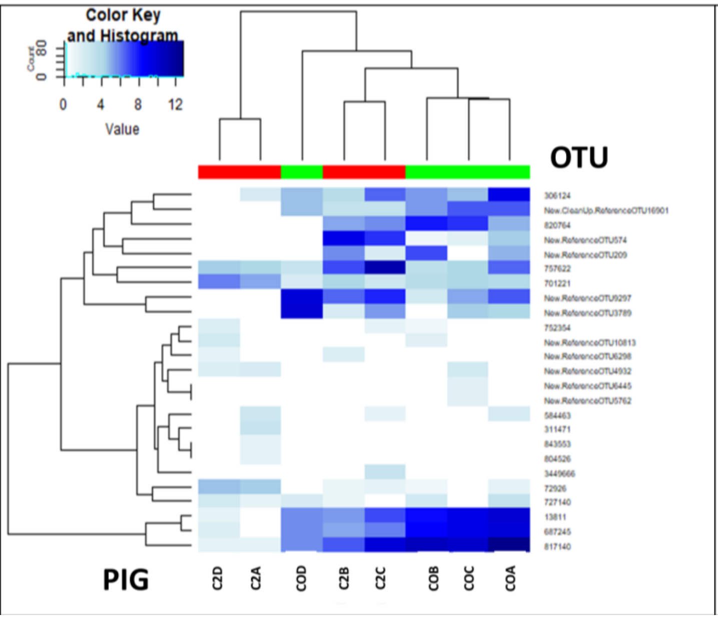

Figure S1b

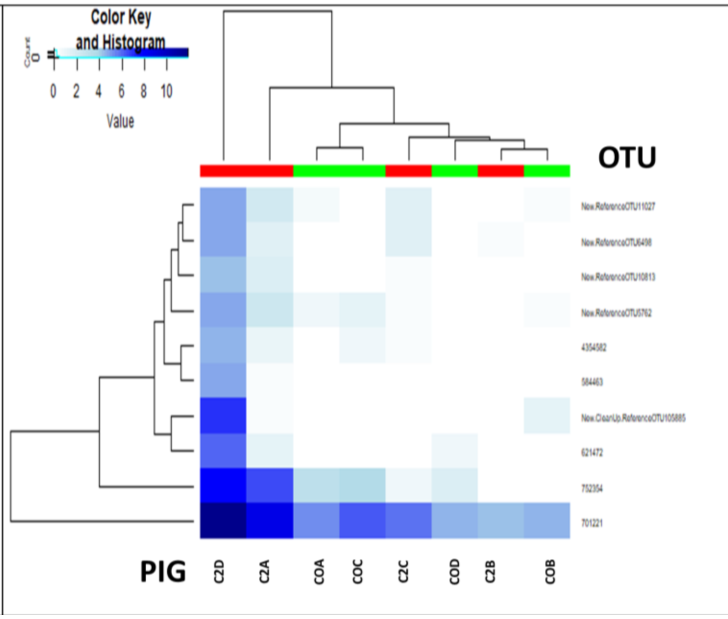

Supplementary Figure S1. *Salmonella* Typhimurium infection affects the relative abundance of OTUs at the ileum content and faeces. Heatmap illustrating the mean relative abundance of multiple OTUs at the ileum content (Figure S1a) and faeces (Figure S1b) of non-infected control pigs (green) and Day 2 p.i. infected pigs (red). White colour indicates low abundance while dark blue high values of abundance. Dendrogram was built using hierarchical cluster analysis with Bray-Curtis dissimilarity indices.

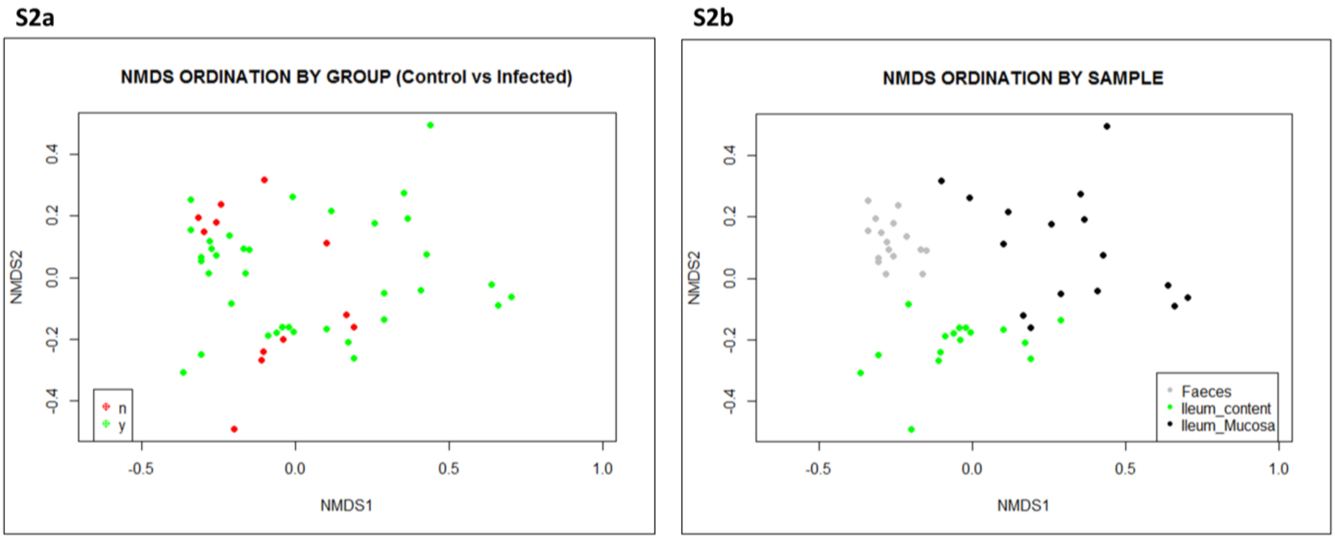

Supplementary Figure S2. Ordination analysis of the functions predicted by (PICRUSt) in samples obtained through the time-course of a *S.* Typhimurium infection in pigs. Figure S2A represents the ordination using Bray-Curtis dissimilarity index and plot by Non-metric Multidimensional Scaling (NMDS) of samples split by infection group. Figure S2B represent Bray-Curtis dissimilarity by NMDS of samples by type of sample factor.

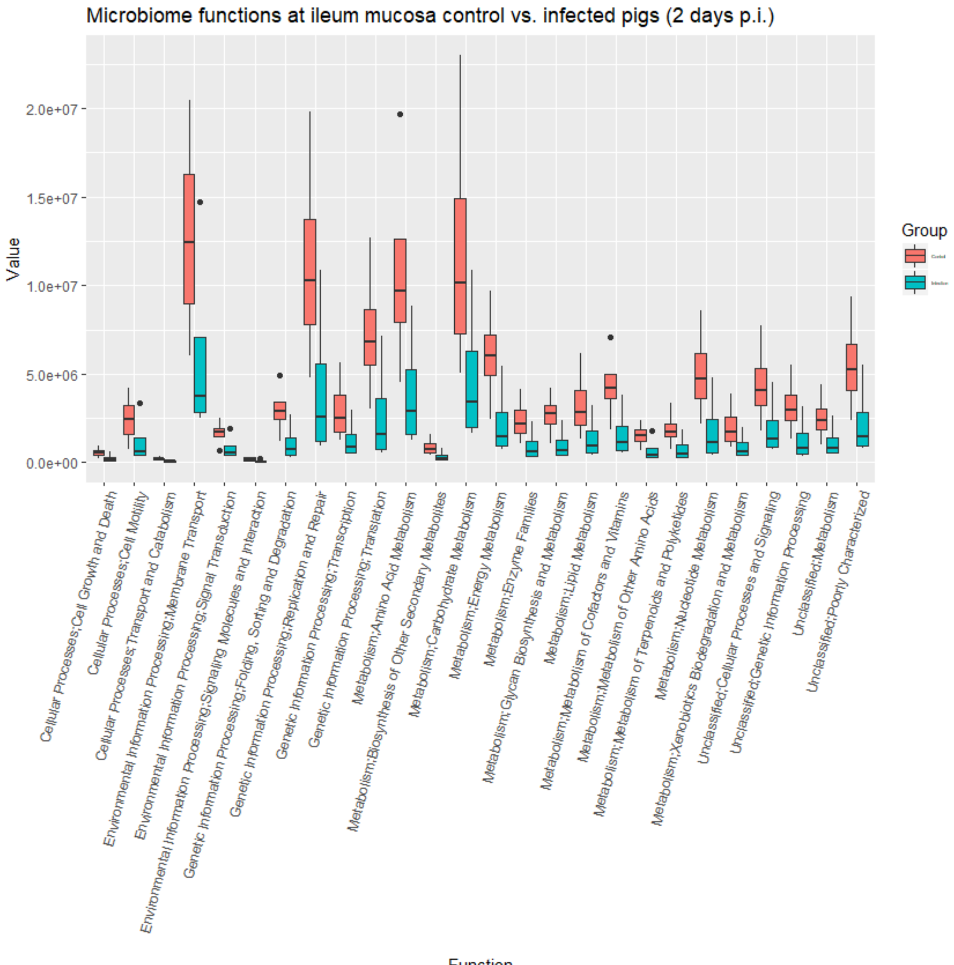

Supplementary Figure S3. Boxplot representation of logarithm of abundance of detected KEGG level 2 functions in the ileum mucosa from control pigs (non-infected) and S. Typhimurium infected pigs at 2 days post infection. Significant differences are expressed by (*) in those functions with a *p*<0.05).
